# Integrated Resuscitation Strategies in Orthopedic Trauma: A Systematic Review of Outcomes of Cardiopulmonary Resuscitation (CPR), Hemorrhage Control, and Damage Control

**DOI:** 10.7759/cureus.93592

**Published:** 2025-09-30

**Authors:** Kshitij Srivastava, Rao Junaid Saleem, Rewant Singh, Abdullah Elrefae, Miqdad Qandeel, Mirza Arsalan Baig, Safeer Ahmad Javid, Muddasir Reyaz Hassan, Muhammad Rizwan Umer, Nadia Malik

**Affiliations:** 1 Trauma and Orthopaedics, Northwick Park Hospital, London, GBR; 2 General and Colorectal Surgery, Northwick Park Hospital, London, GBR; 3 Department of Trauma and Orthopaedic, AlBashir Hospital, Amman, JOR; 4 Trauma and Orthopaedics, Central Middlesex Hospital, London, GBR; 5 Trauma and Orthopedic, London Northwest University Healthcare NHS Trust, London, GBR; 6 Trauma, Royal Sussex County Hospital, Brighton, GBR; 7 Trauma Surgery, Royal Sussex County Hospital, Brighton, GBR; 8 Medicine, National Medical Centre (NMC), Lahore, PAK

**Keywords:** cardiopulmonary resuscitation, damage control orthopedics, early total care, hemorrhage control, massive transfusion protocol, orthopedic trauma, pelvic stabilization, resuscitative endovascular balloon occlusion of the aorta, tranexamic acid, trauma

## Abstract

Orthopedic trauma is a major cause of global morbidity and mortality, often resulting from high-energy mechanisms such as road traffic accidents, falls, and interpersonal violence. Early deaths are frequently due to hemorrhage, coagulopathy, and physiologic instability. This systematic review, following PRISMA 2020 guidelines, included six studies with a total of 28,549 patients. The majority came from the CRASH-2 randomized controlled trial (20,211 bleeding trauma patients, many with orthopedic injuries). The remaining five studies together contributed 8,338 patients to evaluate integrated resuscitation strategies: trauma-specific CPR, hemorrhage control (tourniquets, pelvic stabilization, massive transfusion protocols, tranexamic acid, resuscitative endovascular balloon occlusion of the aorta (REBOA)/embolization), and fracture fixation timing (damage control orthopedics vs early appropriate care/early total care). Evidence indicates that early hemorrhage control, physiologically guided resuscitation, and timely operative intervention improve survival, reduce complications, and optimize functional outcomes. Standardized, protocol-driven approaches and resource-sensitive adaptations remain essential for effective orthopedic trauma care.

## Introduction and background

Trauma continues to be a leading worldwide health challenge and is responsible for millions of deaths and long-term disabilities each year. Current estimates suggest that more than 4 million people die annually from injuries, with road traffic accidents contributing nearly one-third of these fatalities. Among people aged 5 to 29 years, trauma ranks among the top causes of death worldwide. The burden is especially heavy in low- and middle-income countries, where limited resources amplify its impact, and men are almost twice as likely to die from injuries as women. Mortality is often clustered in the first hours after injury, either due to severe traumatic brain injury (TBI) or from uncontrolled hemorrhage within the first 48 hours. While advances in trauma care have reduced deaths from bleeding over the past few decades, TBI-related mortality has shown a steady rise, underlining the ongoing need for effective early interventions [1]. This recognition has shifted modern trauma systems toward integrated, protocol-driven strategies that aim to stabilize physiology, control bleeding, and prevent secondary insults. In orthopedic trauma, the stakes are particularly high because pelvic and long-bone fractures can produce massive hemorrhage and exacerbate shock [2]. Current guidelines emphasize a “circulation-first” approach, prioritizing immediate control of compressible bleeding with tourniquets, pelvic binders, or direct pressure [3].

These measures are supported by hemostatic resuscitation, typically using balanced transfusion ratios (1:1:1 of red cells, plasma, and platelets), permissive hypotension in patients without severe TBI, and early administration of tranexamic acid (TXA) within three hours to counteract fibrinolysis [4]. For non-compressible pelvic bleeding, algorithmic care pathways now include interventions such as angioembolization and resuscitative endovascular balloon occlusion of the aorta (REBOA) [5]. Surgical decision-making has also evolved. Traditional “early total care” (ETC) has given way to more individualized approaches based on physiologic stability. Damage control orthopedics (DCO) favors temporary stabilization in unstable patients to avoid overwhelming systemic stress, while Early Appropriate Care (EAC) supports definitive fixation within 36 hours once key resuscitation thresholds are met [6]. In parallel, trauma-specific adaptations of cardiopulmonary resuscitation (CPR) have been shown to improve outcomes by addressing reversible causes, such as hemorrhage, hypoxia, tamponade, or tension pneumothorax, alongside or even before chest compressions [7].

Taken together, these advances highlight the importance of integrated resuscitation in orthopedic trauma. Strategies such as CPR tailored to trauma physiology, early hemorrhage control, hemostatic transfusion practices, the timely use of TXA or REBOA, and careful timing of fracture fixation can significantly influence both survival and long-term recovery. This review synthesizes available evidence on these interventions to determine where benefits are well supported, where uncertainty remains, and how coordinated care pathways may best improve outcomes for patients with orthopedic trauma, including CPR, Hemorrhage Control, and Damage Control.

## Review

Search strategy

This review followed PRISMA 2020 guidance [8]. A systematic search was conducted across PubMed/MEDLINE, Embase, Scopus, and the Cochrane Library from database inception to June 2025. Controlled vocabulary (MeSH, Emtree) and free-text terms were combined to capture studies on integrated resuscitation in orthopedic trauma. The main concepts included orthopedic trauma, traumatic cardiac arrest, CPR, tourniquet, pelvic binder, pelvic stabilization, REBOA, angioembolization, TXA, massive transfusion protocol (MTP), balanced transfusion, DCO, EAC, and ETC. Boolean operators and truncation were applied, and the search strategy was adapted for each database to maximize sensitivity and specificity. In addition to database searching, reference lists of relevant articles and reviews were screened manually. Major trauma registries with orthopedic subsets, including the American College of Surgeons Trauma Quality Improvement Program (ACS-TQIP) and the German Trauma Registry (TraumaRegister DGU), were specifically reviewed to identify additional studies reporting orthopedic trauma outcomes. This approach ensured that both randomized and observational evidence relevant to CPR, hemorrhage control, and fixation strategies in orthopedic trauma were captured.

Eligibility criteria

The eligibility criteria were defined using the PICO (Population/Patient/Problem, Intervention, Comparison, and Outcome) framework [9]. The population included adults and adolescents with orthopedic trauma, such as isolated extremity or pelvic injuries, or polytrauma with major orthopedic involvement. Interventions comprised integrated resuscitation strategies, including trauma-specific CPR, prehospital tourniquets, pelvic stabilization, TXA, MTPs, REBOA or angioembolization, and fixation timing approaches such as DCO, EAC, or ETC. Comparators included standard care, alternative adjuncts, or different timing strategies. Outcomes of interest were mortality, transfusion requirements, physiologic or end-organ measures, limb and orthopedic complications, and hospital or ICU length of stay. Studies were included if they were randomized trials, comparative observational studies, registries, or systematic reviews/meta-analyses in humans, with extractable quantitative outcomes and full-text available in English up to July 2025. Studies were excluded if they were animal or cadaveric research, case reports, uncontrolled case series, editorials, conference abstracts without full data, or investigations of elective or non-trauma orthopedics where orthopedic outcomes could not be separately analyzed.

Study selection

Two reviewers independently screened titles/abstracts; full texts of potentially eligible articles were assessed against criteria. Disagreements were resolved by consensus with a third reviewer as arbiter. Selection followed PRISMA, with reasons logged for exclusions. Reference lists of included studies were checked to capture additional eligible works.

Data extraction

A standardized form captured study design, setting, population, intervention/comparator details, outcomes, effect estimates, and orthopedic-specific signals (e.g., fixation timing, limb salvage). When needed, authors reported adjusted analyses were prioritized over crude estimates. Data were extracted independently by two reviewers with cross-checks for accuracy.

Risk of bias assessment

We applied risk of bias 2 (RoB 2) for randomized trials, ROBINS-I for non-randomized studies, and AMSTAR-2 for systematic reviews/meta-analyses, using published guidance and domain-level judgments (confounding, selection, classification, deviations, missing data, measurement, reporting) [10-12].

Data synthesis

Given the heterogeneity across interventions and study designs, a structured narrative synthesis was conducted using effect direction plots, focusing on key outcomes such as mortality, transfusion requirements, and complications. Priority was given first to randomized controlled trials, followed by adjusted comparative observational studies, and then high-quality systematic reviews or meta-analyses. The findings were integrated within an “algorithmic pathway” framework spanning prehospital care, emergency department management, operative or interventional radiology procedures, and definitive fixation to identify areas of consistent evidence and where clinical equipoise persists.

Results

Study Selection Process

**Figure 1:**
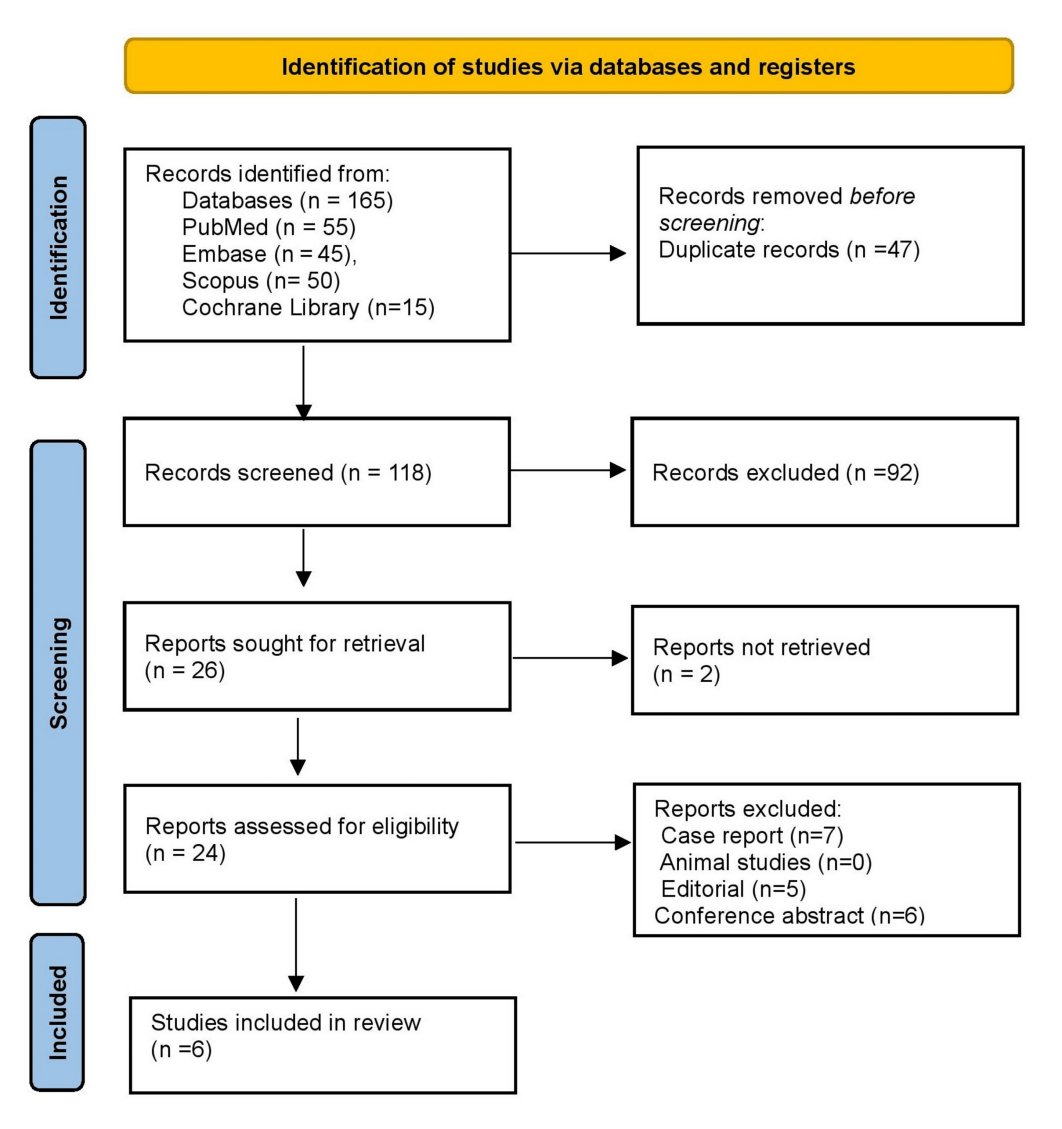
PRISMA 2020 guidance flowchart for study selection process

Figure 1 illustrates the study selection process. A total of 165 records were identified through database searches: PubMed (n=55), Embase (n=45), Scopus (n=50), and Cochrane Library (n=15). After the removal of 47 duplicates, 118 unique records remained for title and abstract screening. Of these, 92 were excluded as not meeting the eligibility criteria. Twenty-six full-text articles were retrieved for detailed assessment; however, 2 could not be obtained. The remaining 24 articles were reviewed in full, leading to the exclusion of 7 case reports, 5 editorials, and 6 conference abstracts. Ultimately, six studies fulfilled all inclusion criteria and were incorporated into the final analysis.

Characteristics of the Selected Studies

Table 1 summarizes the key characteristics of the included studies, outlining author details, study design, population, interventions, and primary outcomes. This overview facilitates comparison of methodologies and findings, highlighting variations in patient demographics, treatment protocols, and outcome measures that may influence interpretation.

**Table 1:** The key characteristics of the included studies TXA: Tranexamic Acid; PH: Prehospital; MTP: Massive Transfusion Protocol; ROSC: Return of Spontaneous Circulation; REBOA: Resuscitative Endovascular Balloon Occlusion of the Aorta; LOS: Length of Stay; EAC: Early Appropriate Care; DCO: Damage Control Orthopedics; ETC: Early Total Care; OR: Odds Ratio; CPR: cardiopulmonary resuscitation

Authors & Year	Population (P)	Exposure/Condition (I)	Comparator (C)	Outcomes (O)	Pathophysiological Findings	Anatomical Impact	Importance
Roberts et al., 2013 [13]	20,211 bleeding trauma patients worldwide (many with orthopedic injuries)	TXA within 3 h	Placebo	↓ all-cause mortality; no ↑ vascular occlusive events overall	Anti-fibrinolysis reduces hemorrhagic death	Applies across extremity/pelvic bleeding	Level-I support for early TXA in trauma resuscitation
Consunji et al., 2020 [14]	3,201 trauma patients across 14 studies	Massive Transfusion Protocols (≈1:1:1–1:1:2)	Pre-MTP care	↓ overall mortality (OR 0.71)	Balanced product resuscitation mitigates coagulopathy	Broad trauma, relevant to high-risk orthopedic hemorrhage	Supports MTP activation pathways
Ko et al., 2024 [15]	4,095 civilian extremity vascular injuries	Prehospital tourniquet	No PH-tourniquet/only in-hospital	↓ mortality (OR 0.48); no ↑ amputation/compartment syndrome	Early compressible hemorrhage control improves survival	Directly pertinent to limb-threatening fractures	Endorses tourniquets in integrated pathways
Vianen et al., 2022 [16]	4,300 traumatic cardiac arrest patients from 38 studies (including blunt/orthopedic trauma)	Trauma-adapted prehospital CPR (thoracotomy, hemorrhage control, airway interventions)	Conventional CPR only	Survival to discharge ≈7%; better outcomes when reversible causes treated	Addresses hypoxia, tamponade, tension pneumothorax, and hemorrhage as main drivers	Includes pelvic/thoracic trauma, often fracture-associated	Shows trauma-adapted CPR improves outcomes vs conventional
Cralley et al., 2023 [17]	407 severe blunt pelvic injuries	REBOA zone-3 vs zone-1	Zone-1	Zone-3 associated with better survival vs zone-1	Distal occlusion may preserve visceral perfusion	Pelvic hemorrhage	Technique nuance matters; if used, favor zone-3 when appropriate
Vallier et al., 2015 [18]	335 polytrauma patients with major fractures	Early Appropriate Care (definitive fixation ≤ 36 h once resuscitated)	Later/less standardized fixation	Fewer complications, shorter LOS	Physiologic stabilization before fixation reduces “second hit”	Axial & femoral fractures	Supports physiology-guided timing (EAC/DCO vs ETC)

Risk of Bias Assessment

Table 2 shows that the risk of bias for the included studies was assessed as follows. Roberts et al. (2013) were rated as low risk using RoB 2 owing to their large sample size, rigorous randomization, and prespecified outcomes [13]. Consunji et al. (2020) and Ko et al. (2024), both systematic reviews and meta-analyses, were judged as moderate risk by AMSTAR-2 due to heterogeneity of included studies and residual confounding despite sound methodologies [14-15]. Vianen et al. (2022), a systematic review and meta-analysis of traumatic cardiac arrest, was also assessed as moderate risk, reflecting strong methodology but limited by heterogeneous designs and predominantly retrospective data in the underlying studies [16]. Cralley et al. (2023), a multicenter retrospective cohort on REBOA in blunt pelvic trauma, was considered at serious risk of bias using ROBINS-I, largely due to confounding by indication and measurement bias in zone selection [17]. Finally, Vallier et al. (2015), a prospective cohort evaluating fixation timing, was rated moderate risk with ROBINS-I, acknowledging some selection bias but strengthened by consistent prospective data and reproducible outcomes [18].

**Table 2:** The risk of bias for the included studies RCT: Randomized Controlled Trial; RoB 2: Risk of Bias 2 tool for randomized trials; AMSTAR-2: A Measurement Tool to Assess Systematic Reviews 2; ROBIS: Risk of Bias in Systematic Reviews; ROBINS-I: Risk Of Bias In Non-randomized Studies of Interventions

Study	Study Design	Risk of Bias Tool	Risk of Bias Rating	Justification
Roberts et al., 2013 [13]	Randomized controlled trial	RoB 2	Low	Large, well-conducted RCT with randomized allocation, prespecified outcomes and analysis; low risk from missing data or selective reporting.
Consunji et al., 2020 [14]	Systematic review/meta-analysis	AMSTAR-2	Moderate	Comprehensive search and meta-analytic methods, but included heterogeneous observational studies of variable quality and had limited GRADE/certainty assessment, lowering overall confidence.
Ko et al., 2024 [15]	Systematic review/meta-analysis	AMSTAR-2	Moderate	Good methodology and pooled observational data; certainty limited by heterogeneity, diverse outcome definitions, and inconsistent adjustment for confounding in component studies.
Vianen et al., 2022 [16]	Systematic review/meta-analysis	AMSTAR-2	Moderate	High-quality systematic review with pooled estimates of traumatic cardiac arrest outcomes; limitations include heterogeneity of included studies and predominance of retrospective designs.
Cralley et al., 2023 [17]	Multicenter retrospective cohort	ROBINS-I	Serious	Confounding by indication (sicker patients more likely to receive REBOA), selection/measurement bias in zone selection, and limited ability to control residual confounders despite a multicenter design.
Vallier et al., 2015 [18]	Prospective cohort/protocol implementation	ROBINS-I	Moderate	Before-after/observational elements and potential selection bias; however, prospective data collection and consistent effect direction increase confidence, though residual confounding remains.

Discussion

Orthopedic trauma remains a major contributor to global morbidity and mortality, particularly in settings where high-energy mechanisms such as road traffic accidents and falls predominate [19]. Patients frequently present with complex injuries complicated by hemorrhagic shock, coagulopathy, and physiologic derangement, necessitating integrated resuscitation strategies that address both immediate survival and long-term functional outcomes. This systematic review examined three major domains of resuscitation in orthopedic trauma: CPR, hemorrhage control, and damage control strategies. Traumatic cardiac arrest (TCA) is associated with poor survival, but outcomes improve when resuscitation is tailored to trauma-specific reversible causes. Conventional CPR alone yields minimal benefit in TCA, as compressions do not address hemorrhage, tension pneumothorax, or tamponade. The Advanced Trauma Life Support (ATLS) protocol, developed by the American College of Surgeons, provides a structured approach to trauma care, emphasizing rapid assessment and intervention. Prehospital care focuses on the primary survey, which includes Airway, Breathing, Circulation, Disability, and Exposure (ABCDE), to identify and manage life-threatening conditions promptly [20].

Vianen et al. (2022) demonstrated that survival to discharge, while low overall (~7%), was significantly higher when interventions such as thoracotomy, airway management, or rapid hemorrhage control accompanied resuscitation [16]. This finding highlights that orthopedic trauma patients with severe blunt or penetrating injuries benefit most from CPR approaches that prioritize circulation and reversible pathology rather than chest compressions alone. Hemorrhage remains the leading preventable cause of early trauma mortality. Our review found strong evidence supporting multiple complementary strategies. The CRASH-2 trial confirmed that TXA given within three hours reduces hemorrhagic deaths without increasing thromboembolic events [13]. Consunji et al. (2020) reported that balanced MTPs significantly improve survival by mitigating trauma-induced coagulopathy [14]. Similarly, Ko et al. (2024) showed that prehospital tourniquet use in extremity vascular trauma reduced mortality without higher rates of amputation or compartment syndrome [15]. For non-compressible hemorrhage, Cralley et al. (2023) demonstrated improved survival with distal (zone-3) REBOA compared to proximal occlusion, supporting its use in select pelvic fracture patients [17]. Taken together, these findings emphasize that early and targeted hemorrhage control, whether via tourniquets, TXA, MTP, or REBOA, is central to successful orthopedic trauma resuscitation.

Fracture stabilization timing represents another critical element of integrated trauma care. Vallier et al. (2015) found that EAC, defined as definitive fixation within 36 hours once physiologic stabilization is achieved, was associated with fewer complications and shorter hospital stays compared to delayed or less standardized approaches [18]. By contrast, DCO remains necessary in unstable patients to avoid the “second hit” inflammatory response of major surgery. Physiologic thresholds such as lactate, base deficit, and pH remain essential in guiding decision-making. These results suggest that while both DCO and EAC have roles, individualized selection based on resuscitation markers is key to optimizing outcomes. Comparing across these domains reveals that integrated strategies, not isolated interventions, also offer the greatest benefit. Trauma-specific CPR provides meaningful survival only when paired with rapid hemorrhage control. TXA, MTP, and REBOA improve survival but must be linked to surgical decision-making that balances early fixation against physiologic readiness. Standardized pathways that coordinate CPR, hemorrhage control, and fracture stabilization create a framework where resuscitation and definitive orthopedic care complement rather than compete with each other.

This review is limited by the heterogeneity of included studies, which span different populations, settings, and study designs. Only one included trial (CRASH-2) was a large-scale randomized controlled study, while others relied on retrospective cohorts or registry analyses. Most studies addressed trauma broadly rather than orthopedic trauma exclusively, limiting the precision of orthopedic-specific conclusions. Additionally, publication bias and differences in institutional resources may have influenced outcomes, particularly for advanced interventions such as REBOA.

Future research should focus on high-quality, multicenter trials that specifically evaluate integrated resuscitation strategies in orthopedic trauma cohorts. Registry-based studies should stratify outcomes by orthopedic injury type and fixation strategy. Further, standardized physiologic thresholds for transitioning from DCO to EAC require validation across diverse patient populations. Finally, greater emphasis should be placed on long-term outcomes such as functional recovery and quality of life, ensuring that survival gains translate into meaningful rehabilitation for trauma patients.

## Conclusions

Orthopedic trauma remains a significant contributor to global mortality and morbidity, with the greatest burden seen in low- and middle-income countries. This review highlights that integrated resuscitation strategies, combining trauma-specific CPR, early and targeted hemorrhage control, and physiologically guided damage control approaches, are central to improving outcomes. Evidence supports the early administration of TXA, balanced MTPs, prehospital tourniquet use, pelvic stabilization, and selective application of REBOA as effective measures to reduce hemorrhagic mortality. Similarly, surgical timing strategies such as EAC and DCO should be individualized according to physiological stability rather than fixed timelines, thereby minimizing complications and optimizing recovery. While these findings demonstrate meaningful advances, the evidence base remains limited, with most data derived from mixed trauma populations rather than orthopedic-specific cohorts. Future multicenter trials, registry-based analyses, and long-term functional outcome studies are needed to strengthen the evidence for integrated resuscitation pathways in orthopedic trauma and ensure their applicability across diverse health care systems.
